# Antiviral Role of IFITM Proteins in Classical Swine Fever Virus Infection

**DOI:** 10.3390/v11020126

**Published:** 2019-01-30

**Authors:** Cheng Li, Hongqing Zheng, Yifan Wang, Wang Dong, Yaru Liu, Liang Zhang, Yanming Zhang

**Affiliations:** 1 College of Veterinary Medicine, Northwest A&F University, Yangling 712100, China; LiC2018@nwafu.edu.cn (C.L.); zhenghq@nwafu.edu.cn (H.Z.); yaru0913@nwafu.edu.cn (Y.L.); eerduosizl@163.com (L.Z.); 2 Tianjin Customs, Tianjin 300000, China; wyflc1201@163.com; 3 College of Veterinary Medicine, Henan University of Animal Husbandry and Economy, Zhengzhou 450046, China; superdong1990@163.com

**Keywords:** classical swine fever virus, IFITM proteins, endosomes, interferon-stimulated genes

## Abstract

The proteins IFITM1, IFITM2, and IFITM3 are host effectors against a broad range of RNA viruses whose roles in classical swine fever virus (CSFV) infection had not yet been reported. We investigated the effect of these proteins on CSFV replication in mammalian cells. The proteins were overexpressed and silenced using lentiviruses. Confocal microscopy was used to determine the distribution of these proteins in the cells, and immunofluorescence colocalization analysis was used to evaluate the relationship between IFITMs and the CSFV endosomal pathway, including early endosomes, late endosomes, and lysosomes. IFITM1, IFITM2, or IFITM3 overexpression significantly inhibited CSFV replication, whereas protein knockdown enhanced CSFV replication. In porcine alveolar macrophages (PAMs), IFITM1 was mainly located at the cell surface, whereas IFITM2 and IFITM3 were mainly located in the cytoplasm. Following CSFV infection, the distribution of IFITM1 changed. IFITM1, IFITM2, and IFITM3 colocalization with Lamp1, IFITM2 with Rab5 and Rab7, and IFITM3 with Rab7 were observed in CSFV-infected cells. Collectively, these results provide insights into the possible mechanisms associated with the anti-CSFV action of the IFITM family.

## 1. Introduction

Classical swine fever virus (CSFV) causes disease in domestic pigs that affects the livestock industry with serious socioeconomic implications. [1,2]. CSFV belongs to the *Flaviviridae* family and the *Pestivirus* genus [3]. CSFV is an enveloped virus containing positive-stranded RNA that encode a large polyprotein, which polyprotein generates four structural proteins and eight nonstructural proteins through post-translational processing [4,5]. Interferons (IFNs) elicit distinct antiviral activity [6] and induce numerous interferon-stimulated genes (ISGs) that protect the host from viral infection [7]. Although there are many studies of ISGs, only a few studies of ISGs as antiviral effectors against CSFV replication have been published [8,9,10,11,12].

IFN-inducible transmembrane proteins (IFITMs) have been shown to have antiviral effects in viral infections, especially of enveloped viruses such as Ebola virus, influenza A virus, West Nile virus, hepatitis C virus, and dengue virus [13,14,15,16,17]. Studies of biochemical and membrane fusion showed that IFITMs inhibit viral entry possibly by altering the fluidity of cellular membranes [18]. To date, five IFITMs have been identified in humans, including IFITM1, IFITM2, IFITM3, IFITM5, and IFITM10. IFITM5 is limited to osteoblasts, and the function of IFITM10 remains largely unknown [19]. Thus, most studies of IFITMs have mainly been focused on IFITM1, IFITM2, and IFITM3. IFITMs exhibit high amino acid sequence similarity in humans and swine [20,21,22]. Different IFITM family members inhibit viruses with different efficiencies, which are also dependent on the infected cell line [18,23]. Accumulating evidence suggests that localization of IFITMs and their influence on vesicular compartments are closely linked to their antiviral activities [24,25,26]. Human IFITM1 is mainly located at the plasma membrane, whereas IFITM2 and IFITM3 have been reported to be located in intracellular compartments [27,28,29]. However, the location of swine IFITMs in porcine alveolar macrophages (PAMs) remains unclear.

IFITM1 interacts with CD81 and occludin, which are co-receptors of HCV, to disrupt HCV entry [30]. IFITM2 and IFITM3 but not IFITM1 inhibit Rift Valley fever virus by preventing endosome fusion with the virus membrane but have no effect on virus attachment, endocytosis, or replication kinetics [24]. Additionally, IFITM3 has been reported to prevent fusion of the host endosomal membranes and/or plasma membrane with the viral membrane to block enveloped virus entry [23]. Nonetheless, relatively little is known about IFITM-mediated antiviral activity against CSFV. IFITMs are mainly located at the membranes of early endosomes, late endosomes, and lysosomes. Considering that CSFV entry and replication rely on these compartments [31], it would be interesting to investigate whether IFITMs influence vesicular compartments during CSFV infection.

In light of these data and given that IFITMs act as inhibitors in viral infection, we aimed to determine the effect of IFITMs on CSFV replication. In addition, our goal in the current work was to examine the distribution of IFITMs in PAMs and whether IFITMs affected CSFV infection.

## 2. Materials and Methods

### 2.1. Cells and Virus

Human embryonic kidney cells (HEK-293T; American Type Culture Collection [ATCC], Manassas, VA, USA; CRL-11268) were cultured in Dulbecco’s minimal essential medium (DMEM; Gibco, Grand Island, NY, USA) supplemented with 10% fetal bovine serum (FBS; Gibco). Porcine alveolar macrophages (PAMs; ATCC; CRL-2845) were cultured in RPMI 1640 medium (Gibco) supplemented with 10% FBS. The Shimen strain of CSFV was obtained from the Control Institute of Veterinary Bio-products and Pharmaceuticals (Beijing, China). Experiments involving CSFV were standardized according to the Laboratory Biosafety Manual and strictly performed in the P3 biosafety laboratory.

### 2.2. Real-Time Quantitative PCR (RT-qPCR)

Based on the genetic sequences of porcine *IFITM1*, *IFITM2*, *IFITM3*, and *Mx1* (GenBank: JQ315414.1, JQ315415.1, JQ315416.1, and DQ095779.1), specific primers were designed (Table 1). RT-qPCR was performed to define the relative mRNA expression of IFITMs and CSFV. Cells were treated with TRIzol to extract total RNA, which was reversed transcribed into cDNA using the HiScript Q RT Supermix for qPCR (Vazyme, Nanjing, China). RT-qPCR was performed using UltraSYBR Mixture (CWBIO, Beijing, China) according to the manufacturer’s instructions. Relative fold changes in gene expression were normalized against *β-actin* using the 2^−ΔΔ*C*t^ threshold method.

### 2.3. Construction and Transfection of Plasmid

*IFITM1*, *IFITM2*, and *IFITM3* with Flag were inserted into the lentivirus vector pCDH-CMV-MCS-EF1 (CMV) to generate CMV-IFITMs and cloned into pEGFP-C1 to generate C-IFITMs, a vector that expressed the IFITM fusion protein and enhanced green fluorescent protein (EGFP). One pair of short hairpin RNA (shRNAs) directed at *IFITM1*, *IFITM2*, and *IFITM3* and the scrambled shRNA lentivirus (shN) control was designed with the RNAi Designer website (http://rnaidesigner.thermofisher.com/). shRNA was inserted into pCDH-U6-GreenPuro (pCDH-U6). The vectors were transfected into PAMs using Turbofect (Thermo Fisher Scientific, Waltham, MA, USA) according to the manufacturer’s instructions.

### 2.4. Acquisition and Titration of Lentivirus

CMV vector containing IFITMs (CMV-IFITM1, CMV-IFITM2, or CMV-IFITM3) or pCDH-U6 vectors carrying IFITM shRNA lentiviruses (shIFITM) were serially co-transfected into HEK-293T cells using three ancillary plasmids, pGAG, pREV, and pVSV-G, by Turbofect. DMEM with 2% FBS was used to culture the transfected cells for 16 h, and then the medium was replaced with DMEM containing 10% FBS, 4.0 mM L-glutamine (Invitrogen, Carlsbad, CA, USA), 0.01 mM cholesterol (Sigma, St. Louis, MO, USA), 0.01 mM L-α-phosphatidylcholine (Sigma), and 1:1000 diluted Chemically Defined Lipid (Invitrogen) followed by another 48 h of incubation. Three types of lentivirus (IFITM1, IFITM2, and IFITM3) in the cell supernatant were collected by centrifugation at 1500× *g*. Lentivirus titers were estimated in HEK-293T cells. Each of the lentiviruses was inoculated into 10 groups of cells in 96-well plate with a 10-fold dilution series (10^−1^–10^−10^), with the CMV vector or shN used as a negative control. The infected cells were detected with a fluorescence inversion microscope (Nikon, Tokyo, Japan), and positive cells manifested by green fluorescence were counted after culture in DMEM with 2% FBS for 48 h. Lentivirus titers were expressed as 50% tissue culture infectious dose (TCID_50_) mL^−1^.

### 2.5. Establishment and Detection of IFITM-Overexpression and -Knockdown Cell Lines

Four lentiviruses (CMV-IFITMs and shIFITMs), for which titers were sufficiently high for inoculation of PAMs with polybrene (Sigma, St. Louis, MO, USA), were added to the culture medium to enhance the infection rate. The medium was refreshed at 8 h after infection of lentivirus, followed by another 48 h of incubation. Positive cells were selected with puromycin (Thermo Fisher Scientific). Empty CMV and shN served as negative controls. To survey the positive cells that exhibited green fluorescence, a fluorescence inversion microscope (Nikon) was used. The success of overexpression and knockdown of IFITMs was determined by western blot.

### 2.6. Western Blot

Cell samples were mixed with radioimmunoprecipitation assay lysis and extraction buffer and lysed on ice. A small portion of the sample was used for quantitative determination using the BCA Protein Assay Kit (Keygen, Jiangsu, China). Residual protein samples were used for electrophoresis with 12% sodium dodecyl sulfate polyacrylamide gel electrophoresis (SDS-PAGE) and transferred onto polyvinylidene difluoride membranes (Millipore, Burlington, MA, USA). Following blocking in 5% non-fat milk, the membranes were incubated with antibodies including mouse anti-Flag monoclonal antibody (MAb; CWBIO), rabbit anti-IFITM1 polyclonal antibody (PAb; Cusabio, Wuhan, China), rabbit anti-IFITM2/3 PAb (Cusabio), mouse anti-GFP MAb (SUNGENE BIOTECH, Tianjin, China), and mouse anti-β-actin MAb (CWBIO) at 4 °C overnight. After washing, peroxidase-conjugated goat anti-mouse (or rabbit) IgG secondary antibodies (CWBIO) were incubated with the membrane at room temperature for 2 h. Using chemiluminescence buffer, the protein bands were exposed.

### 2.7. Cell Viability Assay

According to the manufacturer’s instructions, cell viability assay in IFITM-overexpression and -knockdown cell lines was performed using Cell Counting Kit-8 (CCK-8) (Beyotime, Shanghai, China).

### 2.8. Virus Titration By Indirect Immunofluorescence Assay (IFA)

The virus titers of CSFV in the cellular supernatant were estimated by IFA. PAMs were inoculated with the supernatant of infected cells in a 96-well plate. Ten groups were prepared with a ten-fold dilution series (10^−1^–10^−10^) and incubated for 24 h or 48 h. The negative control was generated by culture with medium without CSFV. All samples were fixed by 1:1 organic fixing liquid (acetone: methanol) at –20 °C for 20 min and permeabilized by 0.1% Triton X-100 at 4 °C for 20 min. After blocking with 5% skimmed milk, permeabilized cells were probed with positive serum against CSFV. Rabbit anti-pig IgG-fluorescein isothiocyanate (FITC) antibody (Sigma) was used following three washes with phosphate-buffered saline (PBS). Immunofluorescence was detected using a fluorescence inversion microscope (Nikon). Negative controls for background staining levels were prepared with mock cells. The viral titers were expressed as TCID_50_ mL^−1^.

### 2.9. Confocal Immunofluorescence Microscopy

The cells were transfected with C1-IFITM1, -IFITM2, and -IFITM3 in 35-mm^2^ dishes for 24 h. After transfection, cells were infected or mock-infected with CSFV for another 24 h. In subsequent experiments, all the samples were fixed by organic fixing liquid at 4 °C for 30 min followed by three washes. Then, the monolayers were permeabilized by 0.1% Triton X-100 at 4 °C for 20 min. After blocking with 5% skim milk, the monolayers were incubated with anti-Rab5 PAb (Santa Cruz Biotechnology, Santa Cruz, CA, USA), anti-Rab7 PAb (Santa Cruz Biotechnology), and anti-Lamp1 PAb (Abcam, Cambridge, UK), respectively, and probed using an Alexa Fluor 594 AffiniPure goat anti-rabbit IgG (H+L) antibody (Yeasen, Shanghai, China). The cells were stained with DAPI (Beyotime) at 25 °C for 10 min and imaged by laser scanning confocal microscopy (LSM510 META, Zeiss, Oberkochen, Germany).

### 2.10. Statistical Analysis

Student’s *t*-test was used for all statistical analyses. Data are presented as the mean ± standard deviation (SD) of three independent experiments. *p*-values < 0.05 were considered to indicate statistical significance.

## 3. Results

### 3.1. Overexpression of IFITMs Inhibits CSFV Replication in PAMs

**Figure 1:**
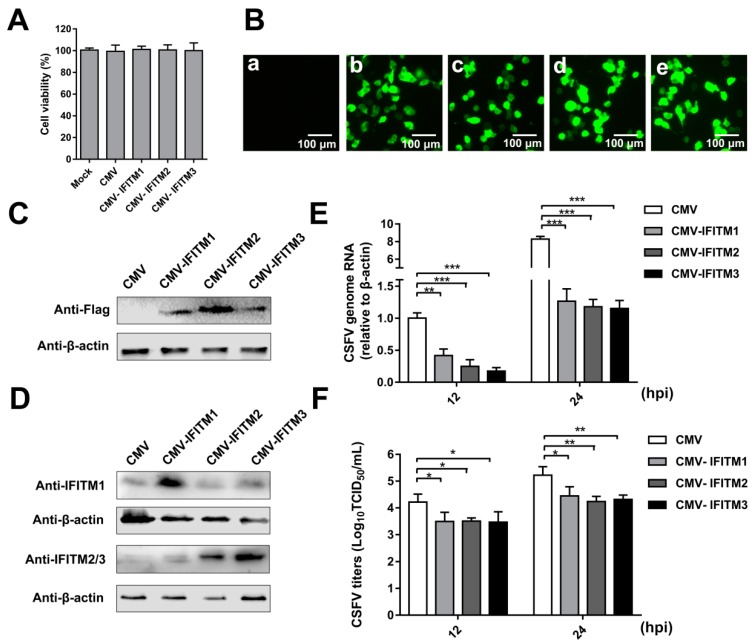
Overexpression of IFITMs inhibits classical swine fever virus (CSFV) replication in porcine alveolar macrophages (PAMs). (**A**) Cell viability of cell lines stably overexpressing IFITMs. (**B**) Confirmation of CMV-IFITM1, CMV-IFITM2, and CMV-IFITM3 lentivirus transfection by detection of enhanced green fluorescent protein (EGFP) reporter. (**a**) Mock-transfected PAMs. (**b**) PAMs transfected with lentiviruses expressing CMV. (**c**) PAMs transfected with CMV-IFITM1 lentivirus, CMV-IFITM2 lentivirus (**d**), and CMV-IFITM3 lentivirus (**e**). Scale bars, 100 μm. (**C**) Western blot analysis of PAM cell lines stably expressing Flag-tagged IFITM1, IFITM2, or IFITM3 or CMV alone using an anti-Flag antibody. (**D**) Western blot analysis of IFITMs in PAM cell lines stably expressing Flag-tagged IFITM1, IFITM2, or IFITM3 or CMV alone using an IFITM1-specific antibody and an IFITM2/3 antibody against IFITM2 and IFITM3, respectively. β-actin served as an internal control. (**E**) CSFV genomic RNA in CMV-IFITM1, CMV-IFITM2, and CMV-IFITM3 cell lines. The CMV, CMV-IFITM1, CMV-IFITM2, and CMV-IFITM3 cell lines were infected with CSFV at a multiplicity of infection (MOI) of 1. CSFV genomic RNA levels were determined by real-time quantitative PCR (RT-qPCR) at 12 and 24 h post-infection (hpi). Data were normalized to *β-actin* expression. (**F**) Infectious progeny viral titers in culture medium from CMV-IFITM1, CMV-IFITM2, and CMV-IFITM3 cells. The viral titers of CSFV in supernatants were quantified by an immunofluorescence assay (IFA) and expressed as tissue culture infective dose (TCID_50_)/mL. Data (**A**,**E**,**F**) represent the mean ± SD of three independent experiments and were measured in technical duplicate. Comparisons between groups were performed by Student’s *t* test. * *p* < 0.05, ** *p* < 0.01 and *** *p* < 0.001.

To analyze the effect of IFITM overexpression on CSFV replication, PAMs that stably expressed IFITM1, -2, or -3 were generated by a lentiviral packaging system. PAMs were transfected with empty plasmid (CMV) as a negative control. The cell viability assay suggested that the growth and viability of CMV-IFITM1, CMV-IFITM2, CMV-IFITM3, and CMV cells were similar to those of PAMs (approximately 100%) (Figure 1A). Green fluorescence in the CMV, CMV- IFITM1, CMV-IFITM2, and CMV-IFITM3 cells was visualized; however, no green fluorescence was observed in the mock-transfected cells under an inverted fluorescence microscope (Figure 1B). Expression of IFITM1, IFITM2, and IFITM3 was detected by western blot using an anti-Flag MAb, whereas no band was detected in the negative control (Figure 1C). Similar results were observed using a rabbit IFITM1-specific PAb and a rabbit IFITM2/3 PAb against IFITM2, which is cross-reactive with IFITM3 (Figure 1D). CMV-IFITM1, CMV-IFITM2, CMV-IFITM3, and CMV cells were incubated with CSFV at a multiplicity of infection (MOI) of 1. CSFV genomic RNA and CSFV titers in the supernatants were evaluated by RT-qPCR and IFA at 12 h post-infection (hpi) and 24 hpi, respectively. As shown in Figure 1E,F, the transcriptional levels and titers of CSFV in CMV-IFITM1, -2, and -3 were significantly lower than those in CMV cells at 12 hpi and 24 hpi. These results suggested that CSFV replication is restricted by overexpression of IFITM1, IFITM2, or IFITM3.

### 3.2. Knockdown of IFITMs Mediated by shRNA Enhances CSFV Replication

**Figure 2:**
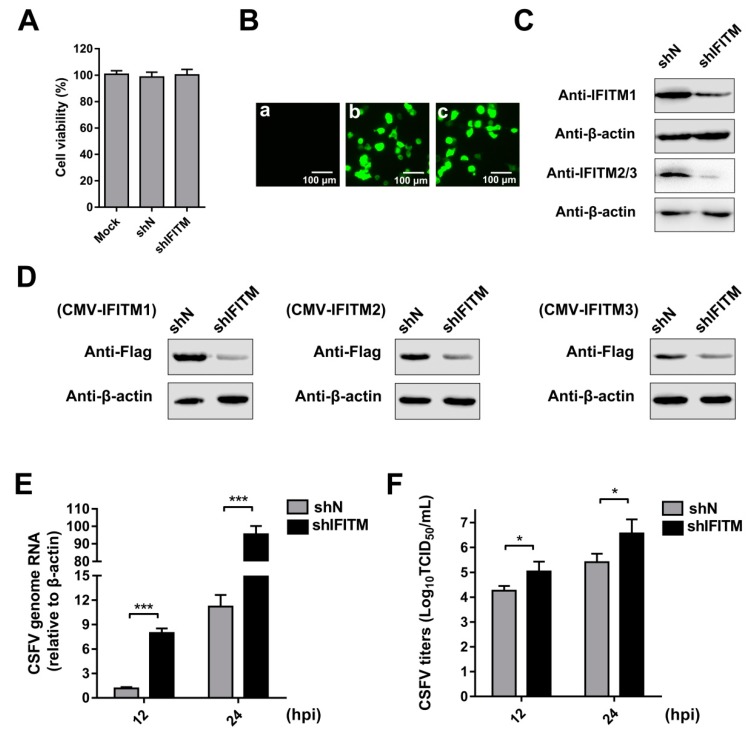
Knockdown of IFITMs mediated by short hairpin RNA (shRNA) enhances CSFV replication. (**A**) Cell viability of stable IFITM-knockdown cell line. (**B**) Confirmation of recombinant IFITM shRNA lentivirus (shIFITM) transfection by detection of EGFP reporter. (**a**) Mock-transfected PAMs. (**b**) PAMs transfected with scrambled shRNA lentivirus (shN). (**c**) PAMs transfected with shIFITM. Scale bars, 100 μm. (**C**) Western blot analysis of IFITM expression in IFITM-knockdown cells using an IFITM1-specific antibody and an IFITM2/3 antibody against IFITM2 and IFITM3, respectively. β-actin served as an internal control. (**D**) Western blot analysis of IFITM expression using an anti-Flag antibody in shIFITM cell lines following transfection with CMV-IFITM1, CMV-IFITM2, or CMV-IFITM3 for 48 h. β-actin served as an internal control. (**E**) CSFV genomic RNA in shIFITM cell lines. The shN and shIFITM cell lines were infected with CSFV at an MOI of 1. CSFV genomic RNA levels were determined by RT-qPCR at 12 and 24 hpi. Data were normalized to *β-actin* expression. (**F**) Infectious progeny viral titers in culture medium from shIFITM cells. The viral titers of CSFV in supernatants were quantified and expressed as TCID_50_/mL. Data (A, E, and F) represent the mean ± SD of three independent experiments and were measured in technical duplicate. Comparisons between groups were performed by Student’s *t* test. * *p* < 0.05 and *** *p* < 0.001.

To directly explore the role of endogenous IFITMs in CSFV replication, we constructed cell lines stably transfected with IFITM shRNA using a lentiviral packaging system. Because IFITM1, IFITM2, and IFITM3 show high amino acid sequence similarity in swine [21,22], we designed one shRNA primer targeting *IFITM1*, *IFITM2*, and *IFITM3*. PAMs were transfected with shIFITM or shN. A cell viability assay was performed in the cell lines. The results showed that the growth and viability of shN and shIFITM cells were similar to those of the control (Figure 2A). Green fluorescence was visualized in shN and shIFITM cells under an inverted fluorescence microscope; however, no green fluorescence was detected in the mock-transfected cells (Figure 2B). To confirm that knockdown by shRNA was successful, we measured protein levels of the three IFITMs. Because we could not find an antibody that can distinguish endogenous IFITM2 and IFITM3, two experiments were performed. Firstly, we examined endogenous expression of IFITMs in mock, shN, and shIFITM cells using an anti-IFITM1 PAb and an anti-IFITM2/3 PAb (Figure 2C). We found that the expressions of IFITM1 and IFITM2/3 were notably reduced in shIFITM cell lines compared with those in shN cells. Furthermore, we detected expression of IFITMs in mock, shN, and shIFITM cells following overexpression of the three IFITMs. As shown in Figure 2D, we found that the shRNA designed to target IFITMs potently downregulated expression of the three IFITMs. shIFITM and shN cells were infected with CSFV at an MOI of 1 at 12 hpi and 24 hpi. CSFV genomic RNA (Figure 2E) and CSFV titers (Figure 2F) in supernatants were significantly enhanced by downregulation of the IFITM protein. Combined with the results described in 3.1, the anti-CSFV nature of IFITM1, IFITM2, and IFITM3 was revealed.

### 3.3. Expression of IFITMs Is Induced by IFN-α Treatment

**Figure 3:**
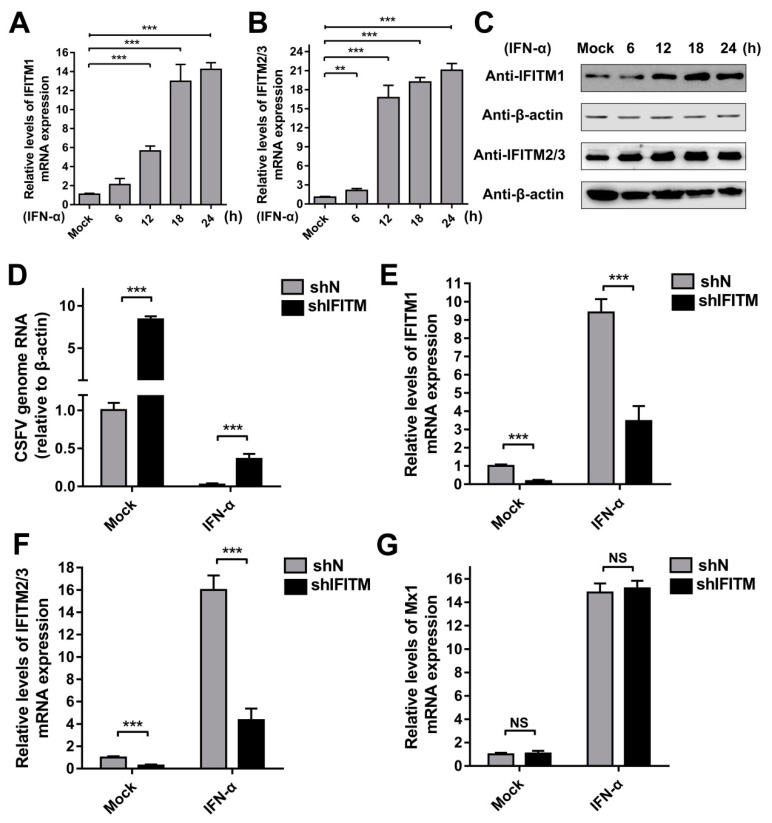
Expression of IFITMs is induced by interferon (IFN)-α treatment. RT-qPCR analysis of *IFITM1* (**A**) and *IFITM2/3* (**B**) mRNA levels (normalized to *β-actin* expression) in PAMs treated with IFN-α (10 ng/mL) at 6, 12, 18, and 24 h post-treatment (hpt). (**C**) Western blot analysis of IFITM expression in PAMs treated with IFN-α (10 ng/mL) at 6, 12, 18, and 24 hpt. β-actin served as an internal control. (**D**) CSFV genomic RNA in shIFITM cell lines with IFN-α pre-treatment. The shN and shIFITM cell lines were pre-treated with IFN-α and then infected with CSFV at an MOI of 1. CSFV genomic RNA levels were determined by RT-qPCR at 24 hpi. Data were normalized to *β-actin* expression. RT-qPCR analysis of *IFITM1* (**E**), *IFITM2/3* (**F**) and *Mx1* (**G**) mRNA levels (normalized to *β-actin* expression) in shN and shIFITM cells. Data (**A**,**B**,**D**,**E**–**G**) represent the mean ± SD of three independent experiments and were measured in technical duplicate. Comparisons between groups were performed by Student’s *t* test. ** *p* < 0.01 and *** *p* < 0.001

To investigate the inducibility of IFITMs in PAMs, we evaluated the expression of IFITMs in the presence of IFN-α. Because the three IFITMs are highly homologous in swine, two specific primers were designed. IFITM1-F and -R were specifically used to detect *IFITM1* gene expression, whereas IFITM2/3-F and -R were used to detect the mRNA levels of *IFITM2* and *IFITM3*. As expected, *IFITM1* mRNA expression was robustly induced by IFN-α at 12 h post-treatment (hpt), 18 hpt, and 24 hpt (Figure 3A). Protein expression of IFITM1 increased under treatment with IFN-α at 12, 18, and 24 hpt (Figure 3C). Similarly, IFITM2/3 expression was significantly induced by IFN-α at both the mRNA and protein level at different time points compared with that in mock-treated PAMs (Figure 3B,C). These results demonstrated that expression of IFITM1, IFITM2, and IFITM3 is induced by IFN-α in PAMs. To evaluate the role of IFITMs in IFN antiviral activity against CSFV replication, shIFITM and shN cells were pre-treated with IFN-α for 24 h followed by CSFV infection. As shown in Figure 3D, the CSFV mRNA level in shIFITM cells was significantly increased compared to that in shN cells with pre-treatment of IFN-α. The results in Figure 3E,F showed that IFITMs expression in shIFITM cells remained obvious decrease even after IFN-α treatment for 24 h. Furthermore, we investigated the expression of Mx1 (another ISG) and found that Mx1 expression did not significantly changed in shIFITM cells (Figure 3G) suggesting that knockdown of IFITMs expression did not effect IFN-pathway and ISG expression.

### 3.4. IFITM Expression is Downregulated By CSFV

**Figure 4:**
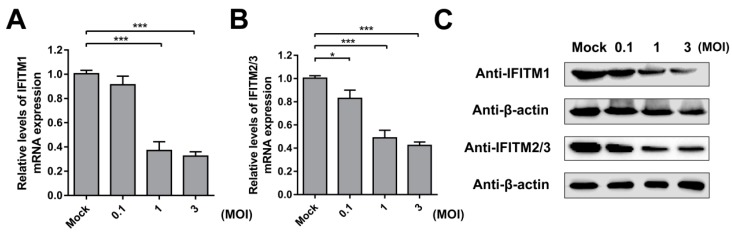
IFITM expression is downregulated by CSFV. RT-qPCR analysis of *IFITM1* (**A**) and *IFITM2/3* (**B**) mRNA levels (normalized to *β-actin* expression) in PAMs infected with CSFV at an MOI of 0.1, 1, or 3 for 24 h. Data represent the mean ± SD of three independent experiments and were measured in technical duplicate. Comparisons between groups were performed by Student’s *t* test. * *p* < 0.05 and *** *p* < 0.001 (**C**) Western blot analysis of IFITM expression in PAMs infected with different doses of CSFV (MOI = 0.1, 1, or 3) for 24 h. β-actin served as an internal control.

Next, we verified the effect of CSFV infection on IFITM expression. PAMs were incubated with different doses of CSFV (MOI = 0.1, 1, or 3) for 24 h. The mRNA and protein levels of IFITM1 were detected by RT-qPCR and western blot, respectively. The results showed that IFITM1 expression was significantly inhibited by CSFV infection at MOIs of 1 and 3 (Figure 4A,C). A similar tendency was observed for IFITM2 and IFITM3 expression. As shown in Figure 4B,C, IFITM2 and IFITM3 expression decreased in CSFV-infected cells at the mRNA and protein level in PAMs with different doses of CSFV compared with mock-infected cells.

### 3.5. Distribution of IFITMs in CSFV-Infected PAMs

**Figure 5:**
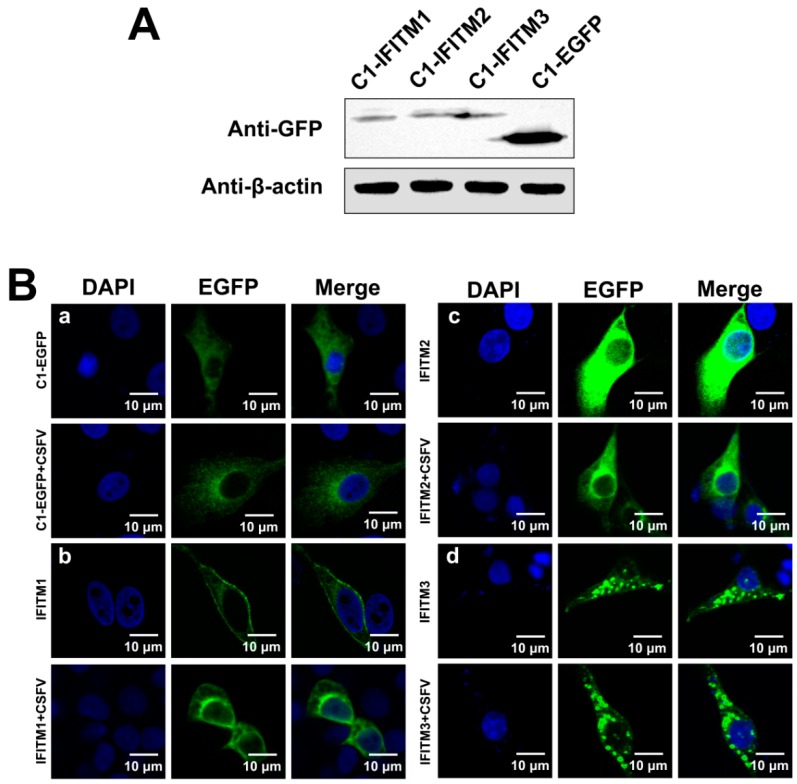
Distribution of IFITMs in CSFV-infected PAMs. (**A**) Confirmation of C1-IFITM1, C1-IFITM2, and C1-IFITM3 transfection in PAMs by western blot detection of fusion proteins with an anti-GFP antibody. PAMs were transfected with C1-IFITM1, C1-IFITM2, C1-IFITM3, or C1-EGFP for 48 h and collected for western blot analysis. β-actin served as an internal control. (**B**) Confocal images of PAMs expressing EGFP-tagged IFITM1, 2, or 3 or EGFP alone. PAMs were transfected with C1-EGFP (**a**), C1-IFITM1 (**b**), C1-IFITM2 (**c**), or C1-IFITM3 (**d**) for 24 h, followed by mock infection or infection with CSFV (MOI = 1) for another 24 h. Cells were fixed and stained with 4′,6-diamidino-2-phenylindole (DAPI, blue). Scale bars, 10 μm.

After we demonstrated that CSFV inhibits IFITM expression, we examined whether CSFV affected IFITM distribution. Because cellular localization of IFITM1, IFITM2, and IFITM3 had not been reported in PAMs, we constructed three plasmids (C1-IFITM1, C1-IFITM2, and C1-IFITM3) with IFITM1, IFITM2, and IFITM3 fused to EGFP to facilitate detection, respectively. Protein expression of IFITM1, IFITM2, and IFITM3 was confirmed by western blot (Figure 5A). PAMs transfected with C1-IFITM1, C1-IFITM2, C1-IFITM3, or empty vector (C1-EGFP) for 24 h were subsequently infected with CSFV (MOI = 1) for another 24 h. The distribution of the IFITM-EGFP protein was observed by laser scanning confocal microscopy. As shown in Figure 5B-b, IFITM1 was mainly distributed on the surface of the mock-infected PAMs with some intracellular localization. In the infected PAMs, IFITM1 appeared to be partially removed from the cell surface. As shown in Figure 5B-c and -d, IFITM2 and IFITM3 was predominantly located in intracellular compartments in the cytoplasm, which did not obviously change in CSFV-infected PAMs. 

### 3.6. IFITMs Do Not Interfere with CSFV Binding But Restrict CSFV Entry

**Figure 6:**
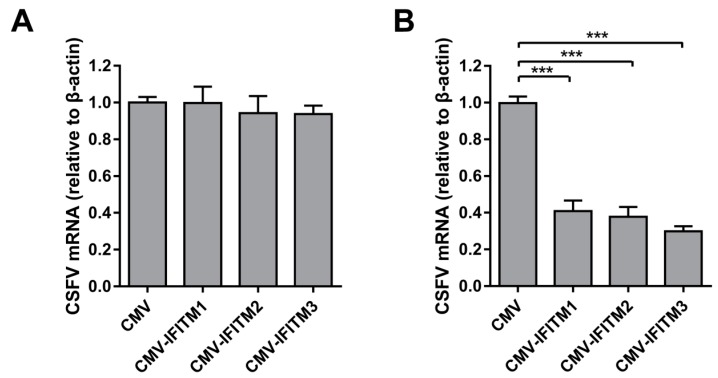
IFITMs do not interfere with CSFV binding but restrict CSFV entry. (**A**) Virus binding assay. PAMs were inoculated with CSFV at an MOI of 1 for 1 h at 4 °C. After washing with ice-cold phosphate-buffered saline (PBS), cell-bound CSFV was quantified by RT-qPCR. Data were normalized to *β-actin* expression. (**B**) Entry assay. PAMs were inoculated with CSFV at an MOI of 1 for 1 h at 4 °C. The viral inoculum was removed, and cells were washed with ice-cold PBS and then incubated with culture medium at 37 °C. After 2 h, PAMs were treated with PBS containing proteinase K to strip the CSFV that remained on the cell surface. CSFV genomic RNA levels were determined by RT-qPCR. Data were normalized to *β-actin* expression. Data represent the mean ± SD of three independent experiments and were measured in technical duplicate. Comparisons between groups were performed by Student’s *t* test. *** *p* < 0.001.

To determine the mechanism of anti-CSFV activity by IFITMs, we examined whether CSFV attachment and entry could be obstructed by overexpression of IFITMs in PAMs, with regard to the successive steps of CSFV infection. To analyze viral binding, PAMs were incubated with CSFV at 4 °C to achieve viral attachment but impair viral entry. The excess virus was then washed away. CSFV genomic RNA collected at this point represented CSFV bound to the cell surface. As shown in Figure 6A, no obvious difference in the amount of bound virus was detected between CMV and CMV-IFITM cells, suggesting that IFITM1, IFITM2, and IFITM3 overexpression did not interfere with CSFV binding. After viral binding, PAMs were incubated at 37 °C to allow CSFV entry. Viral RNA collected at this point represented CSFV entry in the cells. RT-qPCR was subsequently performed with intracellular CSFV genomic RNA. As shown in Figure 6B, CSFV mRNA levels in CMV-IFITM cells were significantly lower than those in CMV cells.

### 3.7. Colocalization of IFITMs with Rab5, Rab7, and Lamp1

**Figure 7:**
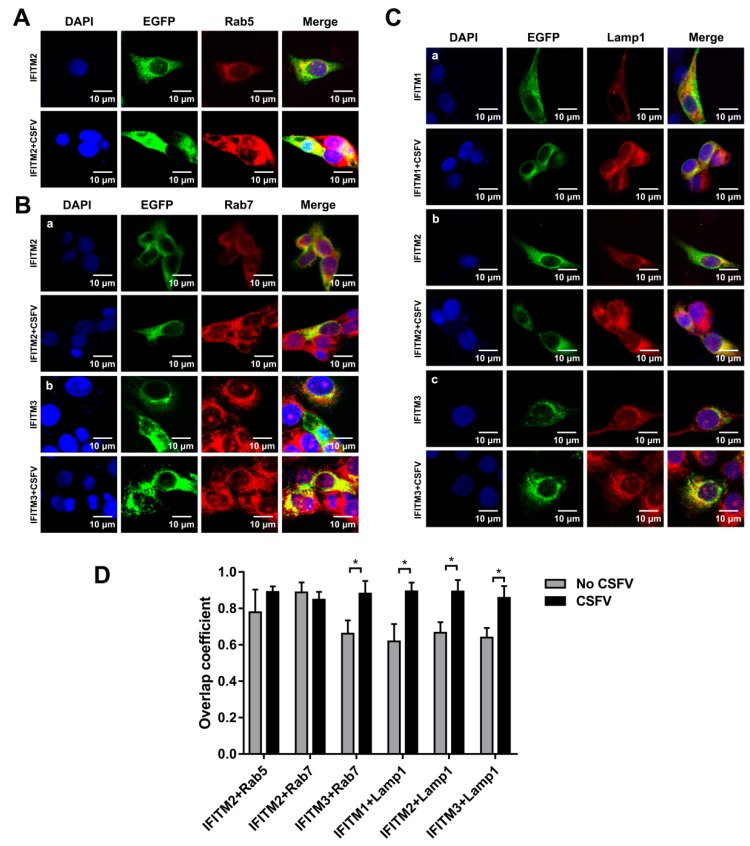
Colocalization of IFITMs with Rab5, Rab7, and Lamp1. Confocal images of IFITM colocalization with Rab5 (**A**), Rab7 (**B**), and Lamp1 (**C**) in mock-infected and CSFV-infected PAMs. The expression of Rab5, Rab7, and Lamp1 was visualized by immunofluorescence staining with an anti-Rab5, anti-Rab7, or anti-Lamp1 antibody, followed by the Alexa Fluor 594 AffiniPure goat anti-rabbit IgG (H + L) antibody. (**A**) PAMs were transfected with IFITM-expressed plasmid for 24 h and then mock-infected or infected with CSFV at an MOI of 1. PAMs were fixed at 24 hpi and stained with anti-Rab5 antibody (red). Nuclei (blue) were stained with DAPI. (**B**) PAMs were transfected with IFITM-expressed plasmid for 24 h, followed by CSFV (MOI = 1) infection for another 24 h. PAMs were stained with anti-Rab7 antibody (red) and DAPI (blue). (**C**) PAMs transfected with IFITM-expressed plasmid for 24 h were infected with CSFV (MOI = 1) for another 24 h. PAMs were stained with anti-Lamp1 antibody (red). Nuclei (blue) were stained with DAPI. Scale bars, 10 μm. (**D**) Overlap quantification of IFITMs with Rab5, Rab7, and Lamp1 was performed using Image J software and measured for individual cells. More than 10 cells were analyzed for each set with statistical tests carried out using Prism. * *p* < 0.05.

It has been reported that CSFV entry into PK-15 cells and PAMs depends on endocytosis. In addition, both Rab5 and Rab7 are required for endocytosis and subsequent productive CSFV infection [31,32]. Given that IFITM restriction of viral infection could be mediated by the endocytic pathway, we hypothesized that overexpressed IFITMs may colocalize with endosomal compartments to interfere with CSFV infection in PAMs. PAMs were transfected with plasmids expressing EGFP-IFITM1, EGFP-IFITM2, and EGFP-IFITM3 fusion proteins for 24 h and infected with CSFV (MOI = 1). Immunofluorescence colocalization analysis showed that IFITM2 partially colocalized with Rab5 (Figure 7A) and Rab7 (Figure 7B-a) in mock-infected and infected cells. IFITM3 was partially colocalized with Rab7 in PAMs even following the CSFV infection (Figure 7B-b). Interestingly, IFITM1, IFITM2, and IFITM3 were found to colocalize with Lamp1 in mock-infected and CSFV-infected cells (Figure 7C). The overlap coefficient is shown in Figure 7D. Therefore, these results revealed colocalization of IFITMs with Rab5, Rab7, and Lamp1 during CSFV infection.

## 4. Discussion

Most ISGs promote innate immunity against viral infection and contribute to the antiviral activity of IFNs [33,34]. Among IFITM family members, much more is known about IFITM1, IFITM2, and IFITM3, which are mainly induced by type I IFN. IFITMs have been shown to broadly suppress viral infection, particularly at the point of viral entry. However, IFITMs differ in their breadth and efficiency of viral restriction, and the antiviral mechanism of IFITMs is not fully understood. To date, the antiviral potential of IFITMs is better understood in human and mouse compared with that in other species. Swine IFITMs have been shown to inhibit influenza A virus [22] and swine IFITM3 inhibits non-enveloped foot-and-mouth disease virus infection [35]. In this study, we identified the antiviral activity of IFITM1, IFITM2, and IFITM3 against CSFV. Additionally we identified subcellular localization of IFITMs in PAM cells and found that IFITMs partlycolocalize with endosomal compartments. 

Previous research of the swine IFITM family revealed that IFITM1, IFITM2, and IFITM3 share high amino acid homology [21,22]. Thus, we were unable to design specific shRNA primers for each IFITM. Nonetheless, to determine the role of endogenous IFITMs in CSFV replication, we constructed a shIFITM cell line stably expressing shRNA targeting mRNA of *IFITM1*, *IFITM2*, and *IFITM3*, and the efficiency was analyzed in conditions of IFITM overexpression. We determined that overexpression of IFITM1, IFITM2, and IFITM3 in PAMs inhibited replication of CSFV at the mRNA level, reducing the viral titer. In contrast, CSFV replication was enhanced in lentivirus-mediated stable knockdown of IFITMs.

Although the mechanism of IFITM antiviral activity remains unclear, we confirmed that all three IFITMs influence CSFV entry. These findings are consistent with the inhibition of entry of vesicular stomatitis virus, West Nile virus, and dengue virus by IFITMs [17,36]. Envelope proteins of CSFV mediate viral attachment; for example, CSFV E^rns^ interacts with membrane-associated heparin sulfate or laminin receptor to mediate CSFV attachment [37,38]. Surprisingly, we found that overexpression of IFITM1, IFITM2, and IFITM3 had no effect on CSFV attachment but significantly inhibited CSFV entry. CSFV entry in PK-15 cells depends on dynamin, cholesterol, and clathrin and requires Rab5 and Rab7, but not Rab11 [31]. However, CSFV entry into PAMs by dynamin and cholesterol-dependent, caveolae-mediated and CSFV endocytosis is regulated by Rab5, Rab7, and Rab11 [32]. Rab5 and Rab7 are critical regulators of early endosomes and late endosomes, respectively, and play an important role in regulating endocytic vesicle trafficking [39,40,41,42]. Accumulating evidence indicates that CSFV is transmitted from early endosomes (Rab5-dependent) to late endosomes (Rab7-dependent) and then into lysosomes [31,32]. Rab5 has been shown interact with CSFV NS4B to enhance CSFV replication by facilitating formation of NS4B complexes [43]. In this study, we found that IFITMs partly colocalized with Rab5, Rab7, and Lamp1. The location of IFITM2 and IFITM3 was most noteworthy, as IFITM2 confocal with Rab5 and Rab7, whereas IFITM3 confocal with Rab7. Furthermore, all three IFITMs colocalized with Lamp1. The characterization of the cellular location of IFITM1, IFITM2, and IFITM3 revealed specific localization patterns linked to the endosomal pathway. Thus, we hypothesized that IFITMs inhibit CSFV replication through interfering with the CSFV endosomal pathway.

In viral infections, such as the dengue virus or influenza A virus, IFITM1 at the cell surface or in the early endosomal pathway suppresses virus entry, whereas IFITM3 prevents virus entry in the late endosomal pathway [44,45]. Furthermore, IFITM3 impairs the fusion of late endosome membranes with intraluminal vesicles that contain virions [29]. Some studies have attempted to explain the IFITM-mediated inhibition of virus entry and suggested that IFITMs alter the intramembranous fluidity and bending to prevent virus entry [18,27]. Other researchers suggested that cholesterol in the endosomal membrane is accumulated by IFITMs, which disturbs cholesterol homeostasis, leading to failed virus release into the cytosol [29,46]. Cholesterol depletion by MβCD significantly inhibits CSFV infection suggesting that CSFV infection depends on membrane cholesterol [31]. Colocalization with endosomal compartments, such as that observed in CSFV-infected cells, might be a characteristic relationship with intracellular cholesterol homeostasis [18,23,29,47]. Hence, it is not surprising that CSFV infection could be restricted by these antiviral effectors acting at the endosomal membrane. We speculated that IFITMs may affect cholesterol to interfere with the CSFV endosomal pathway and this requires further experimentation to explore.

Characterization of the distribution of IFITM1, IFITM2, and IFITM3 in PAMs and localization patterns in the endosomal pathway upon overexpression were partly coincident with previous research in human cells, which revealed that IFITM1 is located at the cell surface and in early endosomes, while both IFITM2 and IFITM3 are located in late endosomes and lysosomes [18,25]. In this study, we found that IFITM2 and IFITM3 colocalized with Rab7 and Lamp1. Unlike in human cells, in swine cells, IFITM2 partly colocalized with Rab5, and IFITM1 was largely limited to the cell surface. Additionally, all three IFITMs partly colocalized with Lamp1. This phenomenon is most likely due to overexpression of IFITM protein resulting in protein distribution in highly acidified lysosomes. [25,27,28]. IFITM1 distribution was altered following CSFV infection. However, alteration in the distribution of overexpressed IFITM2 and IFITM3 was not observed. Interestingly, the distribution of swine IFITM proteins did not change during influenza A virus infection compared with that in uninfected cells [22]. 

Taken together, we illustrated an important role for IFITMs in the inhibition of CSFV infection and revealed a link between the IFITM protein family and CSFV endosomal pathway. We postulated that IFITMs localize to distinct membranes and likely modify the membrane structure or alter endosomal physiology to impair viral membrane fusion. This will be the subject of our future studies.

The exact mechanisms of anti-CSFV action by IFITM1, IFITM2, and IFITM3 warrant further elucidation. However, the evidence presented here provides insight into the close relationship between IFITM anti-CSFV action and the endosomal pathway, which is necessary for CSFV replication. Thus, IFITM1, IFITM2, and IFITM3 are considered potential anti-CSFV effectors that protect the host. This study improved our understanding of the antiviral action of IFITMs in CSFV infection and may contribute to the development of new anti-CSFV treatments.

## Figures and Tables

**Table 1:** Primers used in this study.

Primers	Sequence (5′–3′)	Purpose
CMV-IFITM1-F	CGGAATTCTATGATCAAGAGCCAGCACGA	Amplification of IFITM1
CMV-IFITM1-R	CGGGATCCGTAGCCTCTGTTACTCTTTGCG	
CMV-IFITM2-F	CGGAATTCTATGAACTGCGCTTCCCAGC	Amplification of IFITM2
CMV-IFITM2-R	CGGGATCCGTAGCCTCTGTTACTCTTTGCGC	
CMV-IFITM3-F	CGGAATTCTATGAATTGCGCTTCCCAGC	Amplification of IFITM3
CMV-IFITM3-R	CGGGATCCGTAGCCTCTGTAATCCTTTATGAGCT	
CSFV-F	GAGAAGGACAGCAGAACTAAGC	RT-qPCR for detection of CSFV
CSFV-R	TTACCGCCCATGCCAATAGG	
β-actin-F	CAAGGACCTCTACGCCAACAC	RT-qPCR for detection of β-actin
β-actin-R	TGGAGGCGCGATGATCTT	
shN-F	GATCC*GCTTAAACGCATAGTAGGACT***CAAGAG***AGTCCTACTATGCGTTTAAGC*TTTTTG	Negative control of knockdown
shN-R	AATTCAAAAA*GCTTAAACGCATAGTAGGACT***CTCTTG***AGTCCTACTATGCGTTTAAGC*G	
shIFTIM-F	GATCC*GCAAAGAGTAACAGAGGCTA*C**CAAGAG***GTAGCCTCTGTTACTCTTTGC*TTTTTG	Knockdown of IFITMs
shIFITM-R	AATTCAAAAA*GCAAAGAGTAACAGAGGCTAC***CTCTTG***GTAGCCTCTGTTACTCTTTGC*G	
IFITM1-F	TGGCTTTCGCCTACTCCG	RT-qPCR for detection of IFITM1
IFITM1-R	ACAGTGGCTCCGATGGTCAG	
IFITM2/3-F	TCAACATCCGAAGCGAGACC	RT-qPCR for detection of IFITM2 and IFITM3
IFITM2/3-R	GAGTAGGCGAAAGCCACGAA	
C1-IFITM1-F	CGGAATTCATGATCAAGAGCCAGCACGA	Amplification of IFITM1
C1-IFITM1-R	CGGGATCCCTAGTAGCCTCTGTTACTCTTTGCG	
C1-IFITM2-F	CGGAATTCATGAACTGCGCTTCCCAGC	Amplification of IFITM2
C1-IFITM2-R	CGGGATCCCTAGTAGCCTCTGTTACTCTTTGCGC	
C1-IFITM3-F	CGGAATTCATGAATTGCGCTTCCCAGC	Amplification of IFITM3
C1-IFITM3-R	CGGGATCCCTAGTAGCCTCTGTAATCCTTTATGAGCT	
Mx1-F	TCTGTAAGCAGGAGACCATCAACT	RT-qPCR for detection
Mx1-R	TTTCTCGCCACGTCCACTATC	of Mx1

Restriction enzyme sequences underlined. Short hairpin sequences: loop in bold letters and Interference sequence in italics.
